# Biological Cybernetics: 60 years and more to come

**DOI:** 10.1007/s00422-021-00863-z

**Published:** 2021-02-23

**Authors:** Benjamin Lindner, Peter J. Thomas, Jean-Marc Fellous, Paul Tiesinga

**Affiliations:** 1 Division Bernstein Center for Computational Neuroscience Berlin, Humboldt University Berlin, Philippstr. 13, Haus 2, Berlin, 10115 Germany; 2 Department of Mathematics, Applied Mathematics, and Statistics, Case Western Reserve University, 10900 Euclid Avenue, Cleveland, Ohio, 44106 USA; 3 Departments of Psychology ad Biomedical Engineering, University of Arizona, 1503 E University Blvd, Tucson, AZ 85721 USA; 4 Donders Institute, Faculty of Science, Radboud University, Heyendaalseweg 135, Nijmegen, AJ 6525 Netherlands

**Fig. 1:**
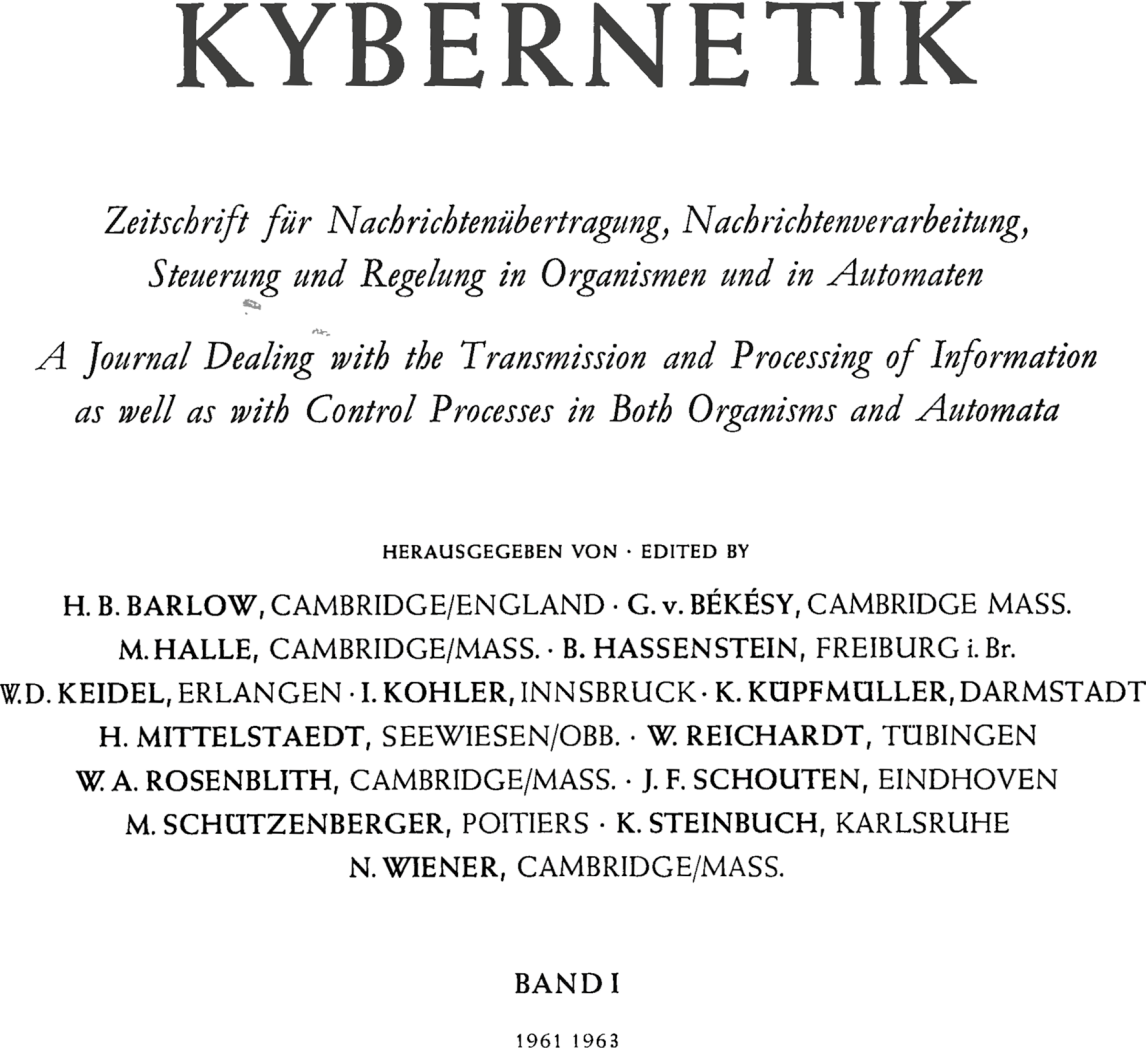
From the title page of the first issue of *Biological Cybernetics* (at that time still *Kybernetik*)

With this February issue, we enter the new year 2021, in which we celebrate the 60th anniversary of *Biological Cybernetics*. The journal was founded under the German title *Kybernetik* in 1961 (see Fig. 1) before the current editors were even born and has been a regular meeting place for theory and experiment in biology in general and neurobiology in particular ever since. We will use this year's occasion to look back at the many pioneering contributions made in our journal. If a positive development of the current pandemic permits, we will also celebrate the journal’s 60th birthday with a real-life symposium in the fall. Of course, we will also take the opportunity to explore future developments in the field of biological cybernetics and to discuss what role the journal can have in supporting them.

This past year, we have (re)opened the journal to new applications in biology, beyond contributions to the field of computational neuroscience. Looking at the articles in this issue, most papers are still devoted to classical problems dealing with, for instance, neuromechanical models, neural network models with plasticity, and models of perceptual inference and motor control. There is one contribution on sensitivity minimization and biological homeostasis that considers as example applications glycolytic oscillations and bacterial chemotaxis—great examples of regulatory systems beyond neuroscience of which we would like to see more in future articles.

Last year, we also started to offer new and original forms of contributions to junior scientists. This initiative has met with enthusiasm, and we publish in this issue the first paper by a junior scientist; more are coming! We see this development as not only an early opportunity to involve young researchers into the writing of journal articles, an essential form of scientific communication, but also into the responsibilities and skills associated with peer review. We hope many graduate students and postdoctoral fellows will contribute as authors or reviewers and help make this initiative a success.

We cannot welcome the reader to 2021 without reflecting on the COVID-19 pandemic, which has taken already the lives of two millions and transformed life for billions more. As a journal that often communicates the results of mathematical modelling of biological systems, we have been encouraged by the extent to which mathematical modelling in biology and medicine has attracted public attention. Despite isolated controversies arising naturally from the sensitivity of model predictions to modelling assumptions and reliable data, the importance of modelling and its predictive power have become widely appreciated by the general public. At the same time, growing public awareness of modelling as an intellectual enterprise highlights the difficulty and the importance of clearly communicating quantitative concepts, such as the basic reproduction number R_0_, or statistical quantities describing the variability of model outcomes, to journalists and other nonspecialists. We hope that with our journal’s review and prospect formats, and, last but not least, with the journal club format of the junior scientist corner, we can modestly contribute to making research results in the field of biological cybernetics more accessible to a broader scientific audience and, by that, indirectly eventually also to the public. In the long run, explanation and education is the only way to defend scientific reasoning, be it in epidemiology, in computational biology, or in neuroscience.

In this issue, we also say farewell to our longest-serving board member, Horace Barlow, who passed away last June at the age of 98. Amazingly, he has been with the journal's editorial board since its foundation in 1961 (cf. Fig. 1). His outstanding contributions in neuroscience are summarized and acknowledged in the obituary by Terrence Sejnowski in this issue.

We close by wishing all of our readers, authors, editors and reviewers a healthy and successful year 2021.

